# Editorial: Universal Health Coverage: The Long Road Ahead for Low- and Middle-Income Regions

**DOI:** 10.3389/fpubh.2021.746651

**Published:** 2021-11-11

**Authors:** Mihajlo Jakovljevic, Chhabi Lal Ranabhat, Mohamed Izham Mohamed Ibrahim, João Paulo Teixeira

**Affiliations:** ^1^Institute of Comparative Economic Studies, Hosei University, Tokyo, Japan; ^2^Department of Global Health Economics and Policy, University of Kragujevac, Kragujevac, Serbia; ^3^Global Center for Research and Development, Kathmandu, Nepal; ^4^Department of Health Promotion & Administration, Eastern Kentucky University, Richmond, KY, United States; ^5^College of Pharmacy, Qatar University, QU Health, Doha, Qatar; ^6^Research Center in Digitalization and Intelligent Robotics (CeDRI), Instituto Politecnico de Bragança, Bragança, Portugal

**Keywords:** universal health coverage, health insurance, health expenditure, health care cost, medical care, health economics, spending and health financing

Universal health coverage (UHC) is one of the core Millennium Development Goals adopted in the United Nations and the World Health Organization's (WHO) strategic agenda. Surprisingly, even as we approach the year 2021, achieving UHC to a reasonable extent and protecting the most vulnerable segments of native populations remains a challenge even for the richest of nations (1).

In recent years, traditional world health sector establishments have assumed that most of the market demand for drugs, medical technologies, and services took place in rich Western societies, including Japan (2). Most of the market supply in terms of innovation and technology production led by multinational businesses, such as Big Pharma companies, also took place in these nations (3, 4). Back in 2000, WHO estimates on national health systems worldwide ranked the top ten systems, seven of which were European and only three of which were Asian (Japan, Oman, and Singapore) (5). Due to urbanization on a mega scale, however, growth in living standards and affordability of medical care and medicines can be seen throughout rapidly developing regions (primarily the BRIC nations (Brazil, Russia, India, China) and Southern and Eastern Asia) (6). In November 2018, a public announcement by the Chancellor of Germany, the largest EU economy, emphasized that most of the essential innovation in this area is now taking place either in North America or Far East Asia, far surpassing the European Union. All of these changes reflect heavily on the market demand for drugs, medicinal devices, services, and long-term care worldwide (7).

Until the early 2000s, the global pharmaceutical market was still heavily dominated by the USA, representing approximately a 4% share of the global population and almost 50% consumption of brand-name medicines expressed in value-based turn over. Japan was ranked second in the same terms, preserving this position for a very long time. The contemporary pace of pharmaceutical innovation remains to be dominated by Western, Japanese, and Israeli-based multinationals (8). On the other hand, demand is exploding among emerging economies and all major investors are aware that the lion's share of growth opportunities as we approach 2050 will take place in these emerging economies, outside of mature high-income Organization for Economic Co-operation and Development (OECD) member nations (10). Emerging markets, such as the BRIC nations or EM7 (BRIC + Indonesia, Mexico, and Turkey), remain the core focus of foreign capital investment in long-term strategies and forecasts (9). The combination of UHC and LMIC presents a paradox. UHC is an ideal health care goal that would see all people around the world have access to all types of health service with financial freedom. LMICs have limited financial, technological, and scientific grounds (1) and structural factors like mind set, governance, management, and policies that could be friendly for UHC (3). Similarly, neither high income countries provide all types of financial and technological support to LMICs nor they (LMICs) adopt easily because the context is totally difference. The context might be political, cultural, service delivery, or the moral ground of service provider and people. Using the aim of UHC, LMIC would strengthen health care delivery systems in a smart way and high-income countries could transfer their technology, health care interventions, and research outcomes without considering profit. Nevertheless, UHC in LMIC seems unlikely unless radical change in structures such as health care delivery issues, supply chain management of health commodities, and human resource management, and in people's mindsets takes place, even if they could get enough funding and technologies from high-income countries.

In addition to a variety of health-economic evaluations and health policy analyses, we also welcome methodological and resource use studies. Health policy considerations relevant to financing mechanisms and health expenditures are welcome, while surrounding issues such as health insurance, reimbursement, and cost-containment strategies will be considered as well. Submissions from academia, industry, and regulatory authorities are strongly encouraged. In exceptional situations like the COVID-19 pandemic, high-income countries could take the initiative to mitigate the public health emergencies by targeting global population who need aid first (4). It demands a healthy debate on a global collaboration, welfare, or social marketing approach for the supply of health commodities other than the free market.

The manuscript submission entitled: “Dynamics of Health Care Financing and Spending in Serbia in the XXI Century” studied Serbia's health care financing system and its contributions to society. The economic recovery, improvement in social policies, and health reform were producing somewhat promising outcomes. Therefore, they are expected to provide a healthcare system that will function optimally and ensure accessibility, affordable and fair services, and a sustainable financing system (Krstic et al.).

The contribution by Ranabhat et al. “Structural Factors Responsible for Universal Health Coverage in Low- and Middle-Income Countries: Results From 118 Countries” found that health financing factors alone are not enough to achieve UHC in LMIC. Health care demand and supply management is not sufficient to achieve UHC; fundamental and structural factors also need to be addressed. Societies in which citizens have an individualistic mindset ultimately create conflict, and a culture of non-transparent corruption and consumption of public resources by the private sector results in poor health delivery. A vicious cycle forms between individualism and health system failure and it is difficult to break it. After the chain breaks in those factors, there is clear path to UHC (11).

Romaniuk et al. have conducted the study: “Health System Outcomes in BRICS Countries and Their Association With the Economic Context.” This study highlights that there is poor association between macroeconomics and health system performance. Core health indicators, like infant mortality, are not associated with GDP per capita. For researchers and policy makers such findings warn that overall economic growth is not sufficient for health care access and equity (12).

The following contribution dealt with comparing macroeconomics and health expenditure indicators of Balkan and Eastern European countries on health and its progress between 1990 and 2014 (13). The findings from Stepovic et al. (14) indicated that these countries have a different trend of global domestic product per capita. Moreover, a significant correlation between health indicators was observed in most of the countries. They recommended medical treatment and pharmaceutical innovations and development with the increasing aging population and non-communicable diseases (Stepovic et al.).

Last but not least, Ranabhat et al. have published another contribution within the Topic entitled “COVID-19 Pandemic: an opportunity for Universal Health Coverage.” It shows that public health emergencies like the COVID-19 pandemic are not only threats but also opportunities to achieve UHC and improve health systems. COVID-19 pandemic response approaches by different countries were not fair and there was more damage due to careless, contradicting, and confusing political decisions. An important lesson could be learnt for possible future public health crises and global collaboration on different levels could be the best strategy (15).

This Research Topic was created in order to address the core challenges of UHC provision in rapidly developing world regions. All of the evolving dynamics in the international medical technology arena will play a big role in creating feasible long-term UHC strategies. Papers published within this Research Topic have largely explored some of these aforementioned and surrounding policy issues across geographic regions and jurisdictions. We believe we may have succeeded in sparking further debate on UHC spread in low- and middle-income countries worldwide.

## Author Contributions

MJ has prepared the manuscript draft while JT, CR, and MM have revised it for important intellectual content. All authors contributed to the article and approved the submitted version.

## Funding

The authors would like to hereby express gratitude for Grant No. 175014 of the Ministry of Education, Science, and Technological Development of the Republic of Serbia, out of which some underlying studies were partially financed. Publication of results was not contingent to the Ministry's censorship or approval.

## Conflict of Interest

The authors declare that the research was conducted in the absence of any commercial or financial relationships that could be construed as a potential conflict of interest.

## Publisher's Note

All claims expressed in this article are solely those of the authors and do not necessarily represent those of their affiliated organizations, or those of the publisher, the editors and the reviewers. Any product that may be evaluated in this article, or claim that may be made by its manufacturer, is not guaranteed or endorsed by the publisher.
